# Musculoskeletal Manifestations in Sjogren’s Syndrome: An Orthopedic Point of View

**DOI:** 10.3390/jcm10081574

**Published:** 2021-04-08

**Authors:** Meletios Rozis, John Vlamis, Elias Vasiliadis, Clio Mavragani, Spiros Pneumaticos, Dimitrios Stergios Evangelopoulos

**Affiliations:** 1 3rd Department of Orhopaedic Surgery, School of Medicine, National and Kapodistrian University of Athens, KAT Hospital, 145 61 Athens, Greece; mrozhs@gmail.com (M.R.); jvlamis@email.com (J.V.); eliasvasiliadis@yahoo.gr (E.V.); spirospneumaticos@gmail.com (S.P.); 2 Department of Physiology, School of Medicine, National and Kapodistrian University of Athens, 157 72 Athens, Greece; kmauragan@med.uoa.gr

**Keywords:** Sjogren’s syndrome, osteoporosis, osteomalacia, joint, coexisting diseases

## Abstract

Sjogren’s syndrome (SS) is a frequent entity with a broad symptomatology spectrum, mainly affecting the salivary and lachrymal glands. The disease also affects the musculoskeletal system targeting bones, specific joints, muscles, and the peripheral nerve system. Disease related clinical manifestations canhave an accumulative impact, as the syndrome is commonly associated with other rheumatic diseases. A literature review was performed with the aim to assess the in-depth association of Sjogren’s syndrome and its treatment agents with the musculoskeletal system and further investigate its potential relevance with common orthopedic postoperative complications.

## 1. Introduction

Sjogren’s syndrome (SS) is a common autoimmune disorder characterized by the lymphocyte infiltration of salivary and lachrymal glands, causing sicca complaints [1,2]. Literature data support that SS may develop either as a sole clinical entity (primary SS) or associated with other autoimmune syndromes [3]. Considering this bipolar incidence, and despite the inability to sharply record the exact rates, SS is grossly estimated to affect 1.3 million American adults and principally women [4].

Central events in SS pathogenesis include the infiltration of salivary and lachrymal glands by activated T and B lymphocytes [5], the intrinsically activated phenotype of salivary gland epithelial cells, as well as the activation of interferon/ B-cell activating factor protein (BAFF) axis [6]. Salivary gland epithelial cells have been shown to orchestrate the local immune response given that they are fully equipped with major histocompatibility complex 2 (MHC-2) and other costimulatory molecules and demonstrate the ability to activate B cells [7,8]. The activation of interferon systems in SS has been long recognized and endogenous nucleic acids have been proposed as major inducers [9,10]. This activation further leads to T cells chemotaxis, resulting in acinar and ductal epithelial cell apoptosis [11,12].

Systemic manifestations of SS involve, among others, the musculoskeletal system. [13]. Besides, the frequent occurrence of SS related complaints in the context of other rheumatic diseases involving the musculoskeletal system such as rheumatoid arthritis (RA) and Systemic Lupus Erythematosus (SLE), highlights the need for a better understanding of the total impact of SS on bone metabolism and other musculoskeletal clinical manifestations from a tight corner of the orthopedic point of view [14].

**Figure 1:**
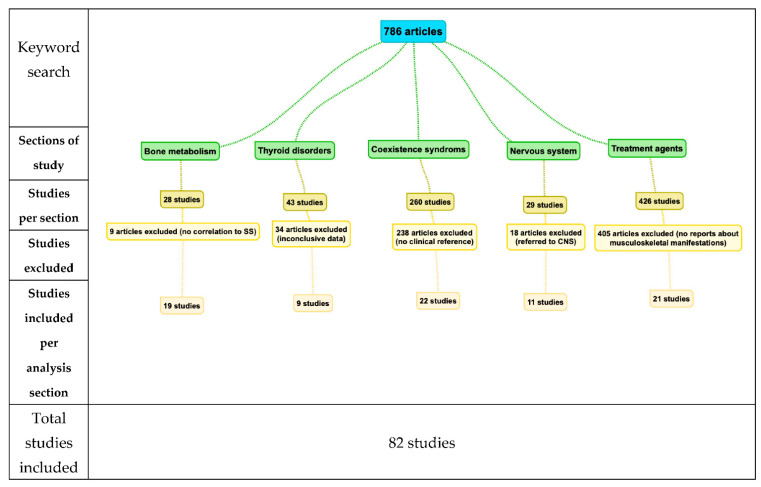
Flowchart of Study analysis.

A literature review was conducted in MEDLINE and EMBASE databases with the following searching terms: Sjogren’s syndrome, osteoporosis, osteomalacia, joint, coexisting diseases (i.e., RA). The inclusion criteria consisted of studies directly associating SS with manifestations from the musculoskeletal and peripheral nervous system. The initial keyword search provided 786 articles, from which 28 referred to bone metabolism, 260 to coexisting syndromes, 29 to nervous system manifestations, and 43 papers to thyroid disorders. We excluded nine articles from the metabolism section as they did not directly connect to SS. Regarding the joint manifestations and coexistence studies, we included 22 papers that referred to the clinical manifestations of both primary SS and SS associated with other diseases. From the 29 studies with reports on the nervous system, 11 studies on PNS were included. Finally, 21 of 426 studies dealing with treatment agents and potential musculoskeletal system’s manifestations and postoperative orthopedic complications were analyzed (Figure 1).

## 2. Discussion

### 2.1. Primary Sjogren’s Syndrome (pSS) and Its Relationship to Bone Metabolic Diseases

Primary SS has the potential todevelop systematic manifestations that entangle the musculoskeletal system, mostly involving the bony, synovial, and cartilaginous tissue, leaving the muscular system unscathed through a general motive of chronic, neuropathic myalgia [15]. This, in addition to high percentages of thyroid disease in SS patients, genuinely implicates the metabolism process of bone and cartilage, which should be further analyzed.

#### 2.1.1. pSS and Osteoporosis

Clinical manifestations of pathological bone metabolism include various well-studied diseases, including osteoporosis and osteomalacia. SS has been implicated inboth of theseconditions through an unclear alteration in the normal signaling pathways. While the SS targeting RANK/RANKL/OPG is under early investigation, its relevance with the Wnt/b-catenin pathway gets evident attention [16].

Two primary receptors regulate the intracellular signaling in the Wnt/b-catenin pathway, the FZD, and LRS5,6 [17]. In a study on the genetic relationship of Wnt receptors with pSS, Fernandez-Torres et al. reported a high possibility of SS development in patients with an A Allele of LRP5 rs606889 and a G FRZB rs409238 allele [18]. Other studies have also shown that pathological values of both sclerostin and DDK1, essential mediators in the Wnt/b-catenin pathway, are related to osteoporosis in patients with pSS [19].

Whether those findings directly correlate SS with osteoporosis and osteomalacia in pathophysiological terms is currently unknown; nevertheless, their direct clinical association should not be ignored. Pasoto et al. performed a study to investigate SS impact on bone mass density (BMD) [20]. Patients with primary SS had lower BMD values in both the hip and lumbar spine than the healthy control group, while in the primary SS group, patients with vertebral fractures showed significant cortical deterioration in pQCT, compared to pSS patients without fractures. However, the fact that all pSS patients were under treatment with corticosteroids at the time of the study cannot identify a primary causative factor for osteoporosis in this case. It is clear though that fragility fractures account for up to 8.5% in patients with SS, and the duration of the disease (*p* < 0.001), patient’s age, and the anti-La/SSB antibodies (*p* < 0.03) are independent factors of osteoporosis development [21].

#### 2.1.2. pSS and Osteomalacia

Osteomalacia is a disorder resulting from the impaired mineralization of bone matrix [22]. Renal function is directly connected to this regulation and is, in turn, hindered in patients with pSS who develop osteomalacia either by tubulointerstitial nephritis or distal renal tubular acidosis (dRTA) [23,24]. Of interest, increased urine pH, indirectly reflecting the presence of tubulointerstitial involvement has been identified as an independent determinant of impaired bone health in a Greek pSS cohort [19]. Several studies highlight the impact of dRTA in patients with primary SS, as it can be the earliest manifestation [23,25,26,27,28,29]. In general, the prevalence of dRTA is 5% and 25–33% for the complete and incomplete disease, respectively [29,30]. Patients with osteomalacia are susceptible to pseudo-fractures. Thus, identifying this entity in patients with pSS is crucial, as early treatment can protect them from further orthopedic interventions [31]. A study by Both et al. reported interestingly different results regarding the mineral density in patients with pSS and dRTA [32]. The investigators ascertained that there was no difference in BMD between pSS patients with and without dRTA. More precisely, these patients had slightly higher BMD, a finding that was hypothetically attributed to hydroxychloroquine (HCQ) intake.

Even though the exact mechanism of pSS actions to bone metabolism has not yet been reported, an indirect association is evident. However, it is not clear which is the main factor interfering with osseous physiology, the SS itself, or the treatment medication and remoter manifestations on target-organs regulating the calcified tissue.

### 2.2. Primary Sjogren’s Syndrome (pSS) and Peripheral Joints

pSS directly impacts peripheral joints, causing mainly arthralgia to 96% of the patients and arthritis to 1.8%. The rates of arthritis have been reported to up to 70% [33,34]. This pain arising from joint involvement predominantly affects the upper extremities, especially the metacarpophalangeal (MCP) joints, causing non-erosive synovitis [35]. The clinical features mimic the RA joint targets, albeit, in pSS, the sacroiliac joints can be involved, and the articular surface is far less commonly damaged with erosions [36]. Since synovitis is the predominant feature, the standard radiological assays may delay pSS diagnosis. For this reason, Riente et al. performing ultrasounds in patients’ hands with pSS, reported that 41.6% had early signs of synovitis in the MCP joints [37]. Similar rates are recorded in another retrospective study where MCP joint arthritis was reported in 51% of pSS patients treated with corticosteroids [38].

The MCP joints are not the only ones involved; knees, ankles, shoulders, and metatarsophalangeal joints can present with pain in a patient with pSS [39]. Although clinical manifestations are symmetrical, mild, and short-lasting, many studies have shown a fluctuation regarding the presentation of initial symptoms [40]. In a late study by Fauchais et al. [41], articular involvement was the initial symptom in 17% of patients and followed the diagnosis in 31.4%.

Another important factor linked to the arthritic manifestations of SS is the anti-citrullinated protein antibodies (ACPAs). ACPAs have been closely linked to RA as a prognostic biomarker for the severity of the disease. In a prospective study by Payet et al. [42], patients with pSS were divided into two teams depending on if they were ACPAs positive or negative. This study showed that patients with pSS and positive ACPAs were significantly predisposed to arthritis development. Those articular manifestations might distinctly differentiate from RA. Simultaneously, up to 42% of patients with pSS reported arthralgia, while arthritis was radiologically confirmed in 18% of them who were—interestingly—positive for ACPAs [43]. Patients with pSS and ACPAs (+) developed non-erosive arthritis through the dominant effect of synovitis at a statistically significant rate (*p* < 0.001), in contrast to pSS-ACPAs (−) patients [44]. Similar results were also validated in another study where ACPAs (+) patients with pSS had not only significantly greater rates of arthritis (*p* = 0.003) but higher frequencies of RA development as well [45].

Finally, arthralgias appear simultaneously with the sicca symptoms in 50% of patients in association with anti-Ro/SSA anti-La/SSB antibodies [46].

Arthralgias along with arthritis can compromise the quality of life and therefore requires either pharmacological treatment or surgical interventions. While arthritis in pSS mostly involves the small peripheral joints, the ankle and the knee joints are not spared, and their treatment is most commonly surgical joint reconstruction. Reviewing the literature, we found only one study investigating the impact of pSS in patients having total knee and total hip arthroplasty [47]. This study found a statistically significant increase in transfusion rates in pSS patients undergoing hip or knee arthroplasty, even though it is not clear if these patients ended up to joint replacement due to the SS itself.

### 2.3. Sjogren’s Syndrome Associated with Other Diseases

Fibromyalgia (FM) is a frequently encountered syndrome that is characterized by chronic widespread pain, fatigue, sleep disturbances, and many other symptoms that impair the quality of life. Altered immune regulation is believed to play a pivotal role in the pathogenesis of FM, as auto-immunity triggers are among the most frequent events preceding its onset. In a recent study of Applbaum et al., the authors found a link between autoantibodies and FM, as one-third of its FM patients with sicca syndrome and/or xerostomia tested positive for Sjögren’s syndrome biomarkers, and the majority of these were also positive for one or more tissue-specific autoantibodies [48]. Additionally, Jeong et al. reported the detection of anti-dense fine-speckled 70 antibodies in FM patients, with significantly higher levels being found specifically in patients with arthralgia and sleep disturbances [49]. Torrente-Segara et al., in their study on 437 pSS patients, reported a 14.6% of FM prevalence. FM-pSS patients demonstrated significantly more constitutional, fatigue and arthralgia symptoms, splenomegaly, genital, skin and ear involvement and dyslipidaemia, while no differences were observed in laboratory markers, imaging techniques or histologic inflammatory findings [50].

While the arthritic manifestations of pSS are mild, SS associated with other diseases has an altered behavior; however, it remains unclear whether this is due to the physical evolution of SS itself or the combined effect of the associated disorders. By definition, SS associated with other diseases occurs alongside another autoimmune syndrome, with RA being the closest one in up to 19.5%, even though cases with three coexistent syndromes have been reported in the literature [51]. This phenomenon seems to have an epigenetic relevance that links those diseases, as shown in a meta-analysis by Toro-Dominguez et al., validating this gene-related contiguity [52]. The investigators isolated a sum of 187 over-expressed and 184 under-expressed genes in SS, RA and SLE, all sharing a common locus and link to the interferon-1 related genes. An additional similarity was encountered regarding another central inflammatory mediator, the TNF-a. Ciccacci et al. studying the role of TNF-a inducible protein 3 (TNFAIP3), an inflammation down-regulating gene, reported its pathogenetic role in all three diseases [53].

There is a concern about these combinations’ accumulative arthritic impact, with those rheumatologic entities being pathophysiologically close.

From a temporality point of view, in a prospective study by Harrold et al., SS was diagnosed in 31.2% of patients with a primary RA diagnosis. This patient cohort manifested higher comorbidity rates, mainly infection [54]. In a study on patients with both RA and SS by Yang et al., the authors revealed that RA manifestations preceded in 64.8% of the patients, in 25.7% the two syndromes had synchronous inception, and only 9.5% of patients with SS developed RA in later stages [55]. Weighing the musculoskeletal system’s burden, they perceived that patients with RA and SS had a significantly higher possibility of erosive arthritis. These results were also validated in another study where patients with both SS and RA acquired more severe arthritic changes and higher disease activity scores [56]. Interestingly enough, for patients with RA developing SS in the latter stages, the periarticular erosions do not increase after the SS emergence [57].

Although pSS primarily affects the hand’s smaller joints, causing arthralgia, SS in the context of other rheumatic diseases adopts an alternative motive sharing and enhancing the characteristics of the coexistent rheumatological disease [58]. Early reports of this phenomenon are presented in a paper by Tsampoulas et al. [59]. In this retrospective study, patients with SS had a mean arthritis/stenosis score of 1.43, while the same mean values were at 4.58 in patients with sSS + RA. Similar results were validated by Amezcua-Guera et al., who reported significant differentiation in the erosions profile in patients with pSS and sSS (*p* < 0.0002) [60].

These reports underline the importance of sSS in the articular manifestations. The latter development of RA seems potent to change the clinical image of the disease, and thus the hypothetical interventions needed to improve the quality of patients’ life. From this point of view, the recognition of biomarkers and their relevance to RA development in patients with pSS seem to be an essential step towards a better management of SS.

Thyroid function is directly connected to normal bone metabolism underlying the impact on common orthopedic interventions like joint arthroplasty [61]. In a retrospective study of 4008 patients, the group with hypothyroidism had statistically significant rates of postoperative infections (3.5%) compared to the control group (1.4%) with the same pool of bacteria [62]. TKA patients with thyroid dysregulation had more significant infections, transfusion needs, deep vein thrombosis, and acute renal failure [63].

The pSS has a close correlation to hypothyroidism with increased anti-thyroid antibodies (anti-TPO, anti-TG) [64,65]. Both pSS and hypothyroidism seem to share common pathophysiological characteristics; a CD4+ T-cell infiltration with the same histocompatibility antigens (HLA class 2) [66]. Interestingly, the anti-thyroid antibodies have been found in higher values in patients with pSS than Hashimoto (+) patients [67].

Sun et al. [68] studied the correlation of SS with autoimmune and non-autoimmune thyroid disease. They found that patients with pSS had a significantly higher possibility of developing both types of thyroid disease than SS negative patients, proposing that every patient with pSS should be examined for possible thyroid dysregulation. This relationship was further validated by another study by Foster et al. [69], who reported that functional thyroid irregularities in 1st degree SS relatives were the most common abnormalities revealed in pSS patients. However, literature data support that despite those similarities, the thyroid disease development does not critically change the severity of the SS itself, but indeed gives an altered clinical image compared to patients without thyroid dysfunction [70].

Peripheral nervous system (PNS) pathologies are a common reason for patient admission in an Orthopaedic clinic. Chronic neural pressure can present with painfulparesthesia and in later stages with a motor deficiency [71]. This pressure phenomenon is indirectly connected to chronic small vessel provision compromise, altering conduction velocity values [72].

In SS, PNS is involved mimicking compression neuropathies, most usually of the upper extremities, a phenomenon induced by small vessel vasculitis, rather than from pure focal compression [73]. The prevalence of peripheral neuropathy in SS patients varies at a range from 1.8% [74] up to 27% of the patients, with one-third of them having additional motor function impairment, and of notice, neurological symptoms can precede the appearance of the primary SS symptomatology [75]. Most patients are expected to develop paresthesia and pain before sicca symptoms, which will emerge after the age of 50 [76]. These results were also reported in the study of Delallande et al. [77]. In 81% of patients, neurological symptoms preceded SS symptoms. In the same study, the most common PNS finding was sensory neuropathy in 34.1%, while 8.5% were diagnosed with multiple mononeuropathies, an entity identified to proceed faster in SS patients [78]. Although sensory deficit seems to be the significant neurological manifestation regarding PNS [79], rare cases of motor-dominant neuropathies have been reported in the literature [80]. On the part of general motor impairment, Pereira et al. [81] reported rates of up to 15% while upper limb areflexia was present in 54% of the SS patients. Finally, the spinal cord is not always spared, and the clinician should be aware of the acute transverse myelitis of the cervical level, which is the most common cord manifestation [82].

### 2.4. Treatment-Related Manifestations

Almost all rheumatologic diseases involve the musculoskeletal system in the spectrum of symptomatology. Orthopedic-related complications, though, are not only related to the disease itself but also the treatment approach. In common practice, most SS-related medications need to halt for a period before and after elective Orthopaedic operations as an attempt to eliminate increased infection rates. In a meta-analysis by Qin et al. [83], the TNFA-α polymorphisms were correlated to SS. Hopefully, having growing concerns about the well-recognized adverse effects of anti-TNFα medications, recent SS treatment guidelines have proposed against the use of these agents [84]. Ruling this category out has given space for different medications that can interfere with normal bone metabolism and common Orthopaedic clinical practice.

#### 2.4.1. Typical Treatment Agents

Hydroxychloroquine (HQ) is the first-line treatment in SS [85]. The administration is proposed not only for the sicca symptoms but also for musculoskeletal pain, reinforced by adding either steroids or non-steroid anti-inflammatory agents [86]. Hydroxychloroquine is structurally and mechanistically similar to the class IA antiarrhythmic quinidine, which inhibits voltage-gated sodium and potassium channels, prolonging the QT interval and increasing the risk of torsades de pointes [87]. HQ has been connected to sudden death in a dosage-related manner [88], but generally, it is a safe treatment that does not have any adverse effects on the musculoskeletal system.

Corticosteroids have a well-studied connection to normal bone metabolism, causing osteoporosis and fragility fractures [89]. Their use remains controversial as an isolated treatment in SS, but it is still proposed for short-term administration in arthritic pain [90]. In their study, Fox et al. [91] have failed to prove that steroid supplementation helps to improve SS symptoms. Despite suggesting against regular use, patients with SS, under corticosteroid treatment, may be vulnerable to low-energy fractures.

Methotrexate (MTX) is strongly suggested as an alternative treatment along with HQ [82], and it is connected to improved outcomes respecting dry eyes and mouth [92]. Experimental data on rats have linked the use of MTX to bone loss [93]. Clinically, patients under MTX treatment should be closely monitored as they are prone to postoperative infections [94] and other implant-induced complications due to increased osteoclast function [95].

#### 2.4.2. Second-Line Treatments

The second-line medication spectrum is currently suggested in SS cases that are not responsive to HQ and MTX treatment and include the administration of leflunomide, azathioprine, rituximab, and abatacept [84,89]. While leflunomide seems to have a protective role in bone absorption by downregulating the osteoclast function [96], rituximab and abatacept have a destructive potential.

Rituximab directly affects the inflammatory process by decreasing TNF-α and IL-6 [97] and demonstrates promising results in patients with SS [98]. Nevertheless, this anti-inflammatory effect has created an increasing concern on the susceptibility to increased infection rates. In a national registry study, patients under rituximab treatment had infection rates of 18.1%, with 3.7% of them being crucial [99]. However, those results were not validated by a newer meta-analysis by Shi et al. [100], where the rituximab treatment group showed similar infection rates compared to the placebo treatment group. This study, though, did not include any data on the orthopedic postoperative infection rates, and it is still unclear whether this kind of medication should be altered in cases of emergent or elective operations.

While rituximab is currently considered a treatment option in SS patients, it has failed to adequately control SS systematic manifestations. Abatacept, a CTLA4-Iginitially developed as a competitive inhibitor of CD28/B7 pathway that acts against CD4 + T-cells, better impacts dry eyes and mouth. In a study by Tsuboi et al., the infection rates were similar to those in the RA patient group, especially if combined with systematic corticosteroids [101]. Additionally, it displays a vital role in articular manifestations, providing significant clinical improvement [102]. There are no growing concerns about its use and possible adverse effects on bone metabolism in the literature. More precisely, abatacept seems to have a rather protective role by increasing bone density and is, therefore, a safe treatment option that does not interfere with the orthopedic procedures [103].

## 3. Conclusions

SS has a close interaction with several aspects of musculoskeletal metabolism and function. Notably, clinician should be aware of the syndrome’s high clinical versatility, especially about the chronological order of joint manifestations (prior or after the sicca) and the silent coexistence with other systematic diseases like RA and SLE. On current data, we can accept that synovitis symptoms of the pSS are mild, but SS in association with other diseases has the potential to enhance the severity of the clinical manifestations. Peripheral neuropathies should be included in this concept despite having a different pathophysiological mechanism other than compression. For SS related musculosketal manifestations, conventional and biological disease modifying agentsare implemented. Finally, the high prevalence of thyroid dysfunction in patients with SS should be considered when treating bone metabolic diseases.

## Data Availability

Not applicable.
